# GRNN-based cascade ensemble model for non-destructive damage state identification: small data approach

**DOI:** 10.1007/s00366-024-02048-1

**Published:** 2024-08-21

**Authors:** Ivan Izonin, Athanasia K. Kazantzi, Roman Tkachenko, Stergios-Aristoteles Mitoulis

**Affiliations:** 1 Department of Civil Engineering, School of Engineering, University of Birmingham, Birmingham, B15 2FG UK; 2 Department of Artificial Intelligence, Institute of Computer Science and Information Technologies, Lviv Polytechnic National University, Lviv, Ukraine; 3 Department of Publishing Information Technologies, Institute of Computer Science and Information Technologies, Lviv Polytechnic National University, Lviv, Ukraine; 4 MetaInfrastructure.org, London, UK

**Keywords:** Small data approach, Interdependent output variables, GRNN, Ensemble model, Cascade, Non-destructive, Damage characterisation, Bridges, Artificial intelligence

## Abstract

Assessing the structural integrity of ageing structures that are affected by climate-induced stressors, challenges traditional engineering methods. The reason is that structural degradation often initiates and advances without any notable warning until visible severe damage or catastrophic failures occur. An example of this, is the conventional inspection methods for prestressed concrete bridges which fail to interpret large permanent deflections because the causes—typically tendon loss—are barely visible or measurable. In many occasions, traditional inspections fail to discern these latent defects and damage, leading to the need for expensive continuous structural health monitoring towards informed assessments to enable appropriate structural interventions. This is a capability gap that has led to fatalities and extensive losses because the operators have very little time to react. This study addresses this gap by proposing a novel machine learning approach to inform a rapid non-destructive assessment of bridge damage states based on measurable structural deflections. First, a comprehensive training dataset is assembled by simulating various plausible bridge damage scenarios associated with different degrees and patterns of tendon losses, the integrity of which is vital for the health of bridge decks. Second, a novel General Regression Neural Network (GRNN)-based cascade ensemble model, tailored for predicting three interdependent output attributes using limited datasets, is developed. The proposed cascade model is optimised by utilising the differential evolution method. Modelling and validation were conducted for a real long-span bridge. The results confirm the efficacy of the proposed model in accurately identifying bridge damage states when compared to existing methods. The model developed demonstrates exceptional prediction accuracy and reliability, underscoring its practical value in non-destructive bridge damage assessment, which can facilitate effective restoration planning.

## Introduction

Ageing bridges are nowadays facing significant challenges, because most of them are approaching the end of their design life. At the same time, natural stressors are proven to be significantly aggravated in intensity and frequency due to the climatic changes [1]. As a result, their performance and response to natural hazards, e.g., floods or extreme temperatures, are also affected by the accelerating deterioration of their material properties in severe environments. Even worse, for one of the most common bridge typologies, i.e., the prestressed concrete bridges, deterioration—for instance due to tendon corrosion and/or concrete cracking—is not easily detectable during routine inspections, unless monitoring, engaging expensive, time-consuming, and on many occasions destructive techniques, is utilised. As a result, the poor condition of such critical assets is not noticed early on and hence failures occur without any substantial prior warning, unless excessive deflections, cracking and catastrophic collapse occurs. Thus, there is an emerging need for developing methods to assess the condition and performance of such assets and rapidly matching health monitoring data and measurements with the structural model response parameters to enable informed decision-making response and adaptation planning [2].

Traditional methods for assessing bridge health typically rely on visual inspections and costly, time-consuming monitoring and structural analyses that not always capture effectively latent defects, such as early fatigue cracks, corrosion of reinforcement or local damage [1]. Structural Health Monitoring (SHM) has emerged in civil engineering to track the health state of structures, including bridges [2]. However, and despite the significant advancements in the SHM field, towards improving the resilience of bridges and consequently extent their service life [3, 4] it is nowadays acknowledged that SHM is a very diverse and multidisciplinary domain that requires the synergy of a broad range of experts (e.g., bridge engineers, AI scientists) to unfold its full potential [5].The above is further justified by the size, uncertainties and complexity of the gathered data, that require extensive processing to correlate measured responses with the health state of the investigated asset [6]. Machine Learning has the potential to analyse Big Data to identify patterns, anomalies, and trends that indicate structural health conditions and link those to potential damage levels [7, 8]. Extensive research has been conducted for developing damage identification methods that exploit modal properties of bridges, such as natural frequencies, mode shapes and modal damping obtained through the interaction of passing vehicles and bridge structures [9, 10]. Nevertheless, modal properties may not be sensitive enough to detect local damage [11]. Furthermore, physical interaction with the asset may not always be possible due to its fragile nature or safety concerns [12]. In particular, physical testing of bridges might be a high risk option especially when deterioration is visible. As a result, a methodology is urgently needed to detect damage states of bridges in a reliable, contactless, and rapid manner.

Bridge deterioration leads, in many instances, to excessive deflections, which in many cases have not been predicted during the design stage [1, 13]. Currently, in the literature there are several non-destructive methods available, e.g., in-situ sensors, UAV inspections, and satellite images [14]. These methods can be deployed for monitoring geometric changes of a bridge asset, e.g., deflections and distortions of the deck and foundation settlement. What is still missing, is a method that links such measurements with certain bridge damage levels. This is an important capability gap, because determining the level of damage is fundamental to restoration strategies, which influence costs and the extent of interventions. This paper proposes and validates, by using real data and numerical modelling, a novel method that exploits machine learning algorithms to interpret stand-off (distant) measurable bridge deck deflections. The method is an efficient and cost-effective way to assist engineers with invaluable first-order decisions of the bridge damage level.

One possible way to identify such informative damage levels for an investigated bridge is by (1) developing a dataset of numerically simulated and validated structural responses, e.g., vertical displacements of the deck translated into drifts over the bridge length, which is the most streamlined and representative way to express deflections in a non-dimensional way, for different realisation of the bridge asset (reflecting a spectrum of damage states) and then (2) pairing the numerically evaluated responses, obtained for the bridge realisations for which the damage state is known, with monitored ones. The development of the dataset is achieved in this paper by analysing variations of finite element models of the asset under investigation, considering different plausible damage scenarios, under the assumption that the cause of the deflection is unknown. These scenarios are the result of several failure modes that are identified as dominant e.g., tendon loss, foundation settlement and other similar damage causes. Nevertheless, the problem cannot be solved with traditional Machine Learning (ML) algorithms because they fail to take into account the interdependencies between input parameters, which influence the outcome, i.e., the definition of the damage state [15]. Yet, these interdependencies are inherent to the problem of structural deflections, especially for prestressed bridge decks. The method of this paper takes into account these critical interdependencies. Hence, the main contributions of this paper can be summarised as follows:

1. A tabular dataset was compiled through simulating several plausible bridge damage scenarios, to enable non-destructive bridge damage state identification using ML methods.
2. A new General Regression Neural Network (GRNN)-based cascade ensemble model was developed to solve the non-destructive bridge damage state identification task for the case of predicting three interdependent output attributes, related to the tendon losses in three characteristic locations of the bridge deck, when analysing small datasets. A method of differential evolution was utilised to tune the optimal parameters of the developed cascade model.
3. A validation of the proposed method performance was conducted on a real case-study bridge asset located in Greece, that was found to experience large vertical deck deflections.
4. Through comparisons with several existing methods, the high effectiveness of using the developed cascade model for solving the problem of non-destructive bridge damage state identification using ML methods in the case of small data analysis was demonstrated.

## Materials and methods

### Dataset development and description

**Fig. 1:**
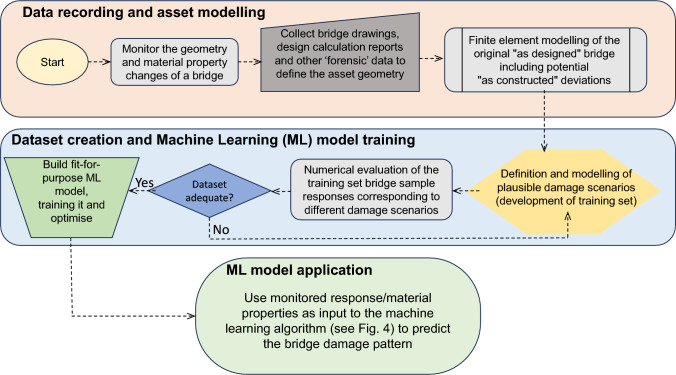
Flowchart of the proposed bridge damage pattern identification methodology

Having identified the deck vertical deflections as the primary damage mode for the bridge asset under investigation, a number of simulated pertinent damage scenarios were considered to account for the different plausible patterns and levels of structural damage. The developed damage scenarios were linked to: (a) realistic scenarios of tendon loss that capture (b) different degrees of tendon area and anchorage losses, as well as the (c) spatial variability of the tendon loss along the bridge deck and (d) the different levels of concrete material degradation [16]. This numerical procedure essentially yields a dataset with several structural response-damage scenario pairs, that can be exploited to train a ML algorithm and subsequently to be utilised for pairing monitored vertical deck drifts with simulated ones. It should be noted that extrapolation beyond the responses that are numerically evaluated is not meaningful due to the non-linearities of the system. Hence, if the monitored responses are outside the response boundaries determined from the samples of the training set the latter should be extended to either cover more damage scenarios or additional failure mechanisms. Ultimately, the procedure aims to link deck deflections to concordant structural damage patterns, see Fig. 1. the overview of the main steps involved in the framework outlined above).

**Fig. 2:**
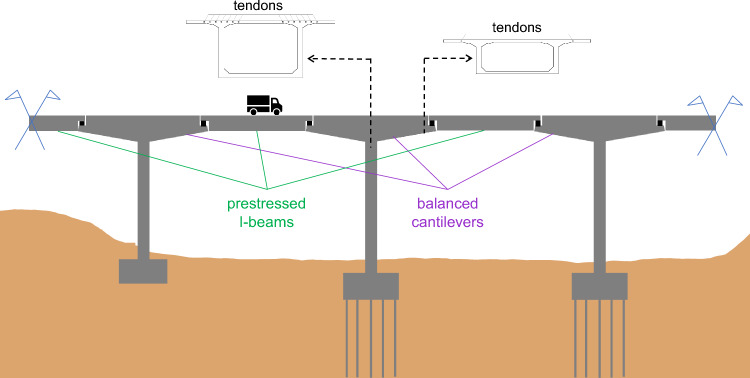
Elevation of the Polyfytos bridge showing part of the structural system with the balanced-cantilevers of box-girder segments and precast simple supported I-beams with precast slabs

To showcase the feasibility of the described method, we have considered as a testbed for the proposed method, a real prestressed-concrete bridge, that is located in the Noth-West part of Greece (see Fig. 2). Although the bridge under study consists of both balanced-cantilever and simple supported precast beam segments, the focus was placed on its balanced cantilever parts. This is because these parts demonstrated excessive deflections, resulting in traffic restrictions and partial closure of the bridge. The observed vertical deflections are under investigation [17], yet the cause of these deflections has not been confirmed because the source of deflections, i.e., the corrosion of prestressed tendons, has not been measured yet, the main reason being the costly interventions needed in this case.

**Fig. 3:**
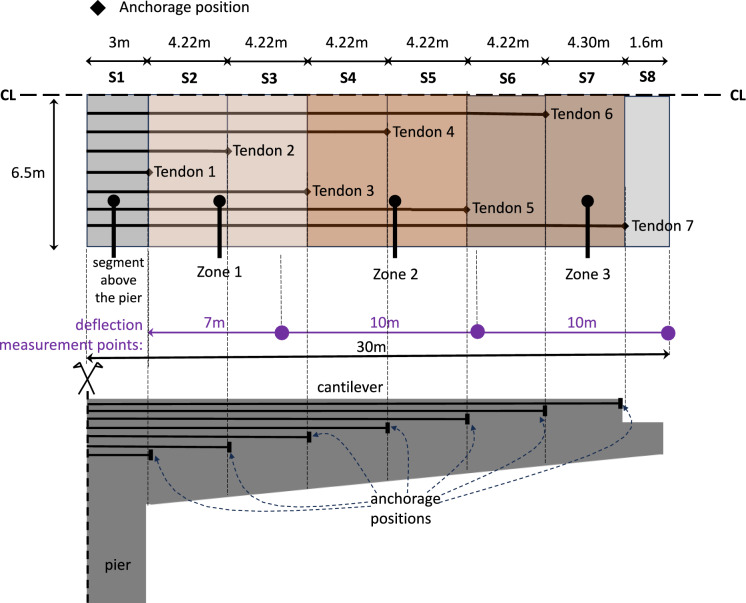
*Above*—plan view of the box-girder deck upper slab showing the number of tendons supporting the eight segments of each cantilever part of the bridge and the anchorage positions. CL indicates that half of the deck width is shown*. Below*: elevation of the pier and the cantilever (not to scale) from the edge of the pier (left) to the end of the deck that supports the precast beams (right). The deflection measurement points are also shown, where the displacement of the bridge deck was measured

The bridge has cantilevers which were casted separately, see in Fig. 2 and segments S1–S7 in Fig. 3, and one segment (S8) that is solid concrete; this is the location of the half-joint connection where the simple supported precast beams are seated upon. The first segment of the deck (S1) is casted directly above the bridge pier and hence it is not of much interest in terms of the observed vertical deflections. This segment is supported by 7 tendon sets, i.e., each set consisting of 14 tendons and each tendon of 7 strands. Segments S2, S3, S4, S5, S6 and S7 are supported by 6, 5, 4, 3, 2 and 1 tendon sets, respectively.

To simplify the problem at hand, but also considering the uncertainty with regard to the anchorages of the tendons, due to lack of access to the original structural drawings at the time of the analysis, the cantilever part of the bridge, in each side of the pier, was subdivided into three Zones, each one consisting of two segments. Hence, as illustrated in Fig. 3, Zone 1 consists of segments S2 and S3 having a total length of 7 m, whereas Zones 2 and 3 consist of segments S4 and S5 as well as S6 and S7, respectively, having a total length of 10 m each. On account that two tendon sets are anchored in each zone, the edge Zone 3 is supported by a total of 4 tendons (i.e., 2 tendon sets) × 7 strands = 28 strands. The intermediate Zone 2 is supported by the 28 strands that are anchored in Zone 3 plus 28 strands that are anchored within the said zone; this condition translates in a total of 56 strands supporting the zone. Consequently, Zone 1, that is close to the pier support, is supported by the strands that are anchored in Zones 2 and 3 and the strands that are anchored directly in the said zone, i.e., 56 + 28 = 84 strands in total.

Based on the description above, it is evident that each zone has different redundancies (ability to redistribute the permanent and variable loads in the case of tendon failures), with Zone 1 being supported by the largest number of tendons, whilst Zone 3 by the smallest. Furthermore, there is a strong interdependency between the zones that determines their structural integrity in case of tendons damage. Thus, any strand damage that occurs in the tendons that are anchored directly in the edge Zone 3 propagates and affects the structural integrity of the preceding Zones 1 and 2. Similarly, any damage that occurs in the tendons that are directly anchored in Zone 2 affects the structural integrity of Zone 1, since the strands of Zone 2 are part of the supporting system of Zone 1. This interdependency between the structural integrity of the zones, poses a unique challenge to the application of any ML methodology. This is because based on a given training set the method should be able not only to predict the damage in the tendons of each zone that can explain the observed vertical deflection, but also to account for the inter-zone interdependencies described above. Thus far, the literature offers no ML methods to addressed such an interdependency.

Table 1 below summarises the 27 tendon loss, cause-agnostic scenarios that were considered for preparing the training set of the case-study bridge. These scenarios are cause-agnostic in that the method is unaware of the deterioration mechanisms that results in the tendon losses, and it therefore focused mainly on the final result, i.e., the damage. This training set forms the basis for the explanation of the monitored vertical deflections in the bridge cantilevers, i.e., pair the monitored vertical drifts with specific damage states, considering the tendon losses as the dominant structural integrity deterioration mechanism. In particular, the training set consists of a total of 27 × 3 = 81 samples, i.e., constituting a relatively small set of data. Each assumed plausible damage pattern for the tendons in each zone, was numerically modelled and analysed. The output of this process are the vertical deflections at the edge of each zone. No movable traffic loading was considered in these damage scenarios. Numerical analyses were conducted for three different concrete properties ranging from soft (cracked) to stiffer (uncracked) deck. The latter is for simulating the effect of the concrete deterioration, by considering variable Young’s modulus i.e., 10, 20, and 35 GPa. The damage scenarios considered are not exhaustive. However, given the complexity of the time-consuming finite element model, the time required for evaluating the responses and to maintain the practicality in the development process of the proposed dataset, these scenarios were carefully selected and they capture a representative spectrum of plausible bridge damage states.

**Table 1:** The damaged strands in bridge zones for the 27 tendon loss scenarios. (analyses were repeated for all three values of concrete Young’s modulus)

Case ID	Zone ID			Case ID	Zone ID		
	Zone 1	Zone 2	Zone 3		Zone 1	Zone 2	Zone 3
*Case 1*	16	0	0	*Case 2*	16	16	0
*Case 3*	16	16	16	*Case 4*	28	12	4
*Case 5*	28	24	16	*Case 6*	28	24	8
*Case 7*	40	24	8	*Case 8*	40	24	16
*Case 9*	40	32	16	*Case 10*	20	0	0
*Case 11*	20	20	0	*Case 12*	20	20	20
*Case 13*	36	16	8	*Case 14*	36	28	20
*Case 15*	36	28	8	*Case 16*	48	28	8
*Case 17*	48	28	20	*Case 18*	48	40	20
*Case 19*	24	0	0	*Case 20*	24	24	0
*Case 21*	24	24	24	*Case 22*	44	20	8
*Case 23*	44	36	24	*Case 24*	44	36	12
*Case 25*	60	36	12	*Case 26*	60	36	24
*Case 27*	60	48	24				

The focus of the present research is to investigate whether a novel ML technique, could obtain reasonable estimates concerning the damage in the tendons of the bridge zones (see Fig. 3), should the vertical deck deflections and the concrete Young’s modulus are known by monitoring and non-destructive investigations. Both inputs, i.e., vertical drifts and concrete Young’s modulus, can be obtained without any destructive testing and interaction with the bridge. The latter is evidence of the significance and practicality of the proposed methodology in real applications.

### Development and optimisation of the General Regression Neural Network

**Fig. 4:**
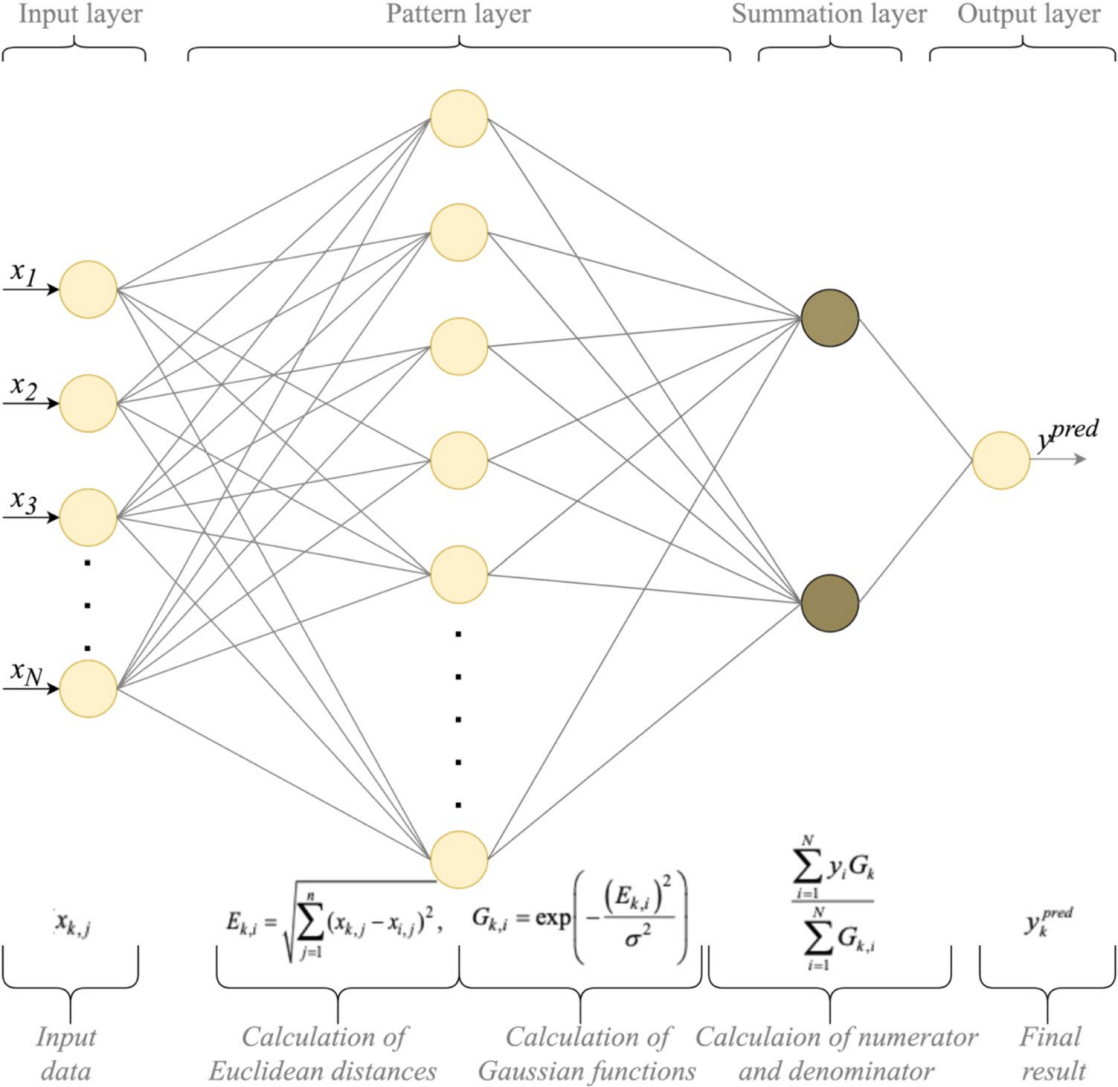
Flowchart of the GRNN topology and main steps for its implementation

The development of the General Regression Neural Network (GRNN) [18] aimed to address the need for an efficient and effective neural network architecture, specifically tailored for regression tasks where the relationship between input and output variables is complex and not easily modelled by linear methods, which is the case of the examined structural problem described before. The topology of the GRNN is illustrated in Fig. 4.

Since its inception, GRNN has found applications in various scientific fields, owing to its ability to approximate continuous functions accurately and efficiently [19, 20]. In particular, GRNN is used in finance, engineering, and medicine for tasks related to, for instance, function approximation, time series prediction, and pattern recognition. The broad utilisation of GRNN could be attributed to the several significant advantages of this neural network against other existing ones. In particular [19, 20]:

- GRNNs, like many other ANNs, are capable of approximating any continuous function with arbitrary accuracy, given enough data and computational resources.
- GRNNs are known for their ability to provide smooth interpolation between training points, resulting in continuous output functions.
- GRNNs employ radial basis functions in the pattern layer to model the relationships between input and output variables. These functions are centered at data points and their influence decreases as the distance from the center increases.
- GRNNs typically operate in a single-pass learning mode, meaning that training the network involves simply storing the training data. This makes training very fast, but it also means that GRNNs do not adapt to data changes once the training is completed.
- GRNNs require the selection of only a single parameter to achieve the maximum prediction accuracy. This minimises the time required for its utilisation and alleviates the need for extensive user expertise.
- GRNNs exhibit superior generalisation properties compared to extant architectures of artificial neural networks. Furthermore, they demonstrate elevated accuracy and rapidity in analysing small datasets.

All these advantages demonstrate the high efficiency of using GRNN for prediction tasks, especially in the case of small datasets.

The following steps summarise the mathematical foundations of using the GRNN. In particular, the implementation procedure of GRNN involves the following steps [19, 20]:

- ***Step 1.*** In the initial GRNN implementation phase, the Euclidean distances ${E}_{k,i}$, between the current vector ${x}_{k,j}$ and each vector ${x}_{i,j}$ within the available training dataset are being determined, using the following formula:
  1 $${E}_{k,i}=\sqrt{\sum_{j=1}^{n}{\left({x}_{k,j}-{x}_{i,j}\right)}^{2}.}$$
- ***Step 2.*** In the subsequent GRNN implementation stage, Gaussian functions ${G}_{k,i}$ are derived from the Euclidean distances ${E}_{k,i}$ that were computed in the preceding step, as delineated by the following formula:
  2 $${G}_{k,i}=exp\left(-\frac{{\left({E}_{k,j}\right)}^{2}}{{\sigma }^{2}}\right).$$
- ***Step 3.*** The final step in the implementation of the GRNN entails the synthesis of the predicted value ${y}_{k}^{pred}$ by means of the following equation:
  3 $${y}_{k}^{pred}=\frac{\sum_{i=1}^{N}{y}_{i}{G}_{k,i}}{\sum_{i=1}^{N}{G}_{k,i}}.$$

The outcomes furnish predicted values for each present vector within the test data sample.

During the application mode, all data containing accessible output attributes can be regarded as training samples, essentially forming the basis for computing the predicted values via utilising Eqs. (1)–(3) for each vector lacking a known output signal. Therefore, regression modelling based on GRNN does not require the use of cross-validation procedures, as the search for the predicted value for a given point triggers a full cycle of GRNN computations (1)–(3), which are performed using all vectors with known output signals.

The GRNN, like any other ANNs [21], has a parameter (in this case, only one) that needs to be tuned. To establish the optimal value of the sole GRNN parameter, i.e., the smoothing factor (σ), the authors employed an optimisation technique that is known as differential evolution [22].

Differential evolution is adeptly suited for global optimisation endeavours, demonstrating proficiency in identifying the global optimum within complex, multimodal, and non-convex search spaces [23]. Its computational efficiency renders it well-suited for optimising intricate functions while imposing a modest computational load. Moreover, its resilience against noise and independence from derivatives of the objective function equip it to effectively navigate noisy or discontinuous tasks [22]. Furthermore, through maintaining a population of candidate solutions, differential evolution concurrently explores diverse regions of the search space, augmenting its capacity to unearth promising solutions [24]. Notably, it accommodates both continuous and discrete optimisation problems, thus presenting versatility across various optimisation domains. Additionally, differential evolution typically exhibits dependable convergence towards near-optimal solutions, even in arduous optimisation scenarios [22, 25]. Overall, the algorithm's implementation simplicity and minimal requirement for parameter tuning facilitate its widespread application across diverse optimisation tasks.

The main steps that are involved in the implementation of the differential evolution optimisation technique are typically the following [22, 24]:

1. Initialisation.
2. Mutation.
3. Crossover.
4. Selection.
5. Population Update.
6. Termination.

To delve into a comprehensive discussion of the matter the optimisation procedure may be conducted using the following steps [23]:

- ***Step 1.*** In the first phase, the generation of an initial population of candidate solutions occurs randomly or using other strategies. Each candidate solution embodies a prospective solution to the optimisation problem.
- ***Step 2.*** A mutation operation is applied to each candidate solution within the population to generate trial vectors. This mutation operation introduces perturbations to the candidate solutions by amalgamating different solutions from the population.
- ***Step 3.*** In the ensuing stage, a crossover operation is employed to produce new candidate solutions through the fusion of the trial vectors with target vectors, representing the original candidate solutions. This operation facilitates the exchange of information between diverse candidate solutions and aids in exploring the search space.
- ***Step 4.*** A comparison between the new candidate solution and the target vector (the original candidate solution) is conducted at this step to ascertain the more promising candidate solution based on an optimization criterion, such as for instance the fitness function value. Subsequently, the superior candidate solution is selected to advance to the subsequent generation.
- ***Step 5.*** Upon the selection of the superior candidate solution, the target vector is replaced with the chosen candidate solution. The iteration of mutation, crossover, and selection steps continues until a termination criterion is met, such as for instance reaching the maximum number of iterations or fulfilling the set convergence criteria.
- ***Step 6.*** The algorithm concludes upon satisfying the termination criterion, typically marked by reaching a predetermined number of iterations or when the improvement in the objective function value falls below a specific threshold.

The procedural approach based on the differential evolution, as elucidated above, is integral for systematically navigating the search space to identify optimal or near-optimal solutions for optimisation tasks. Its efficiency in minimising computational time, renders it particularly well-suited for addressing tasks outlined within academic or practical contexts.

### Novel GRNN-based cascade ensemble model

As discussed in the preceding sections, the peculiarities of the addressed task and the limited available data introduce several challenges regarding the application of artificial intelligence tools in problem-solving.

The first challenge (problem #1) is related to the small size of the training dataset, which imposes several constraints on the application of existing ML methods and artificial neural networks [26]. In the present study, this problem is addressed via using the GRNN, which possesses the highest generalisation properties, has single-pass learning, and unlike other types of ANN (e.g., Multilayer perceptron of Radial-basic Neural Network), demonstrates satisfactory results when analysing limited data volumes [27].

The second challenge (problem #2), is the requirement of predicting three interdependent output attributes (i.e., the tendon losses in each deck zone), each depending on the same set of independent variables (i.e., the vertical drift ratio of Zone 1, the vertical drift ratio of Zone 2, the vertical drift ratio of Zone 3 and the concrete Young’s Modulus). At first glance, such a problem could be tackled using one artificial neural network with three outputs. However, as explored in Xu et al. [28], employing this approach would reduce the accuracy of the obtained results. This is because, during the prediction of each of the three dependent attributes by means of an ANN based on the described approach, the other two will affect the results, and thus significantly decreasing the prediction accuracy. Furthermore, one should also keep in mind the first challenge that was described above. Therefore, to address the second challenge, this study proposes to use three different ANNs to predict the three dependent attributes separately.

The third and most crucial challenge (problem #3) of the investigated task is that the three dependent output attributes that need to be predicted are interdependent. Consequently, on the one hand, the output attributes should not be predicted separately since the predicted outcome will not incorporate all available input information, consequently affecting the accuracy of the predictions [29]. On the other hand, they do not exist for the application mode, particularly to predict each of them considering the other two as additional independent attributes of the given data sample. This is a problem that can be characterised as atypical in ML and in the presented research it is addressed using a new ensemble approach.

**Fig. 5:**
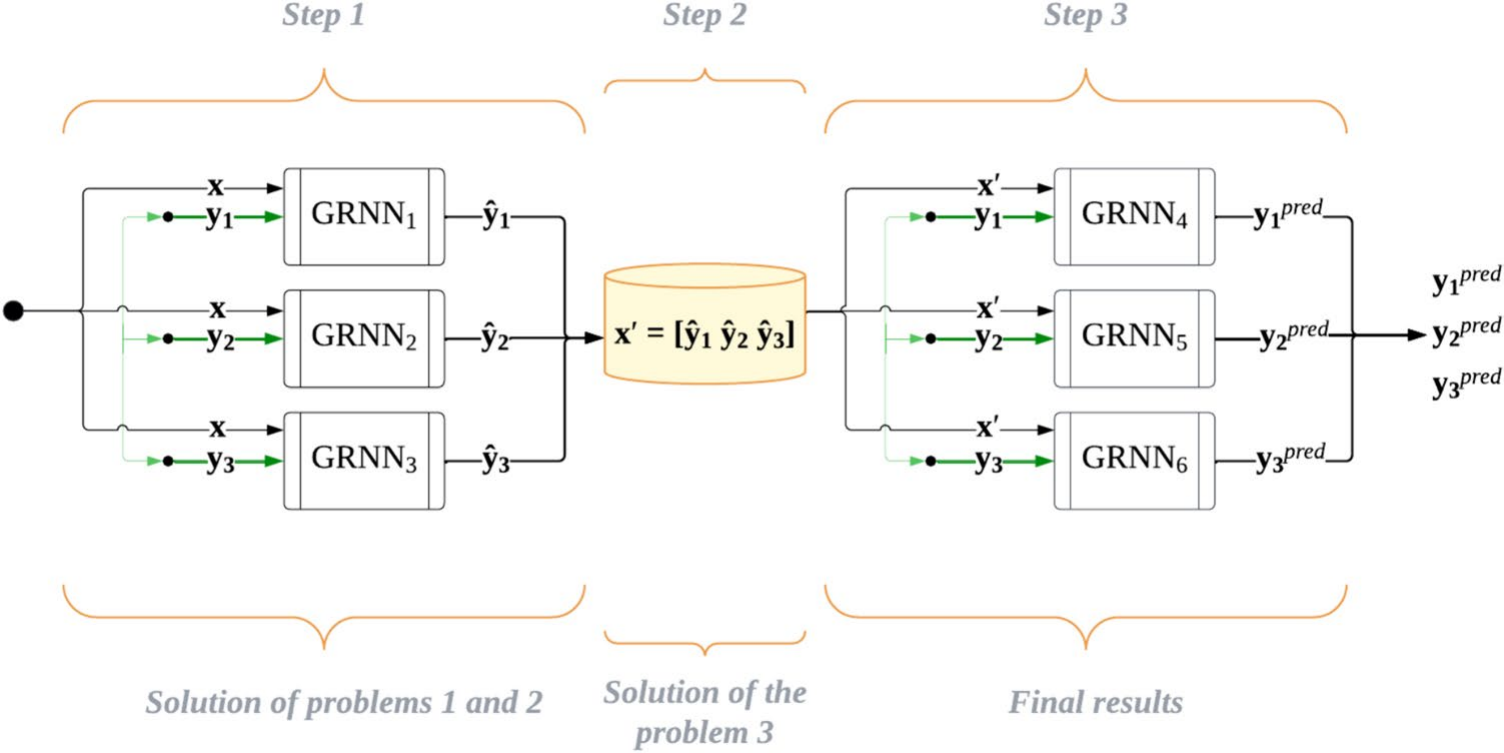
Flowchart of the proposed GRNN-based cascade ensemble model

Taking into account the three aforementioned challenges, this research study proposes for the first time a new cascading ensemble model based on a set of GRNNs for enabling a non-destructive bridge damage state identification. The structure of the proposed model is illustrated in Fig. 5.

As illustrated in Fig. 5, the developed model contains a set of GRNNs that operate in a cascade manner at two levels. Each of the two cascade levels utilises only GRNNs. Thus, problem #1, which is associated with the availability of only a small dataset, is being addressed. At the first cascade level, three different GRNNs are used to individually predict three interdependent attributes. This approach addresses problem #2. Next, the initial dataset is replaced with a new one, where the predicted values of the three sought interdependent attributes act as independent attributes. The initial independent attributes are not considered here (essentially replaced with new ones), and the outputs of this process, as well as at the first cascade level, are the initial output attributes to be predicted. This process essentially addresses problem #3. The final step involves predicting each of the three interdependent attributes separately, based on three different GRNNs but utilising the newly obtained datasets.

The main steps of the algorithmic implementation of the developed GRNN-based cascading ensemble model for non-destructive bridge damage state identification are summarised below:

- ***Step 1.*** Three separate datasets are formed for predicting each of the three dependent attributes individually. It should be noted that the set of independent attributes (i.e., vertical drift ratio of Zone 1, vertical drift ratio of Zone 2, vertical drift ratio of Zone 3 and concrete Young’s Modulus) for all three datasets will be the same. At this step, the prediction is carried out using three GRNNs with the selection of optimal parameters for each one of them using the method of differential evolution. In this case, predicting each of the three attributes separately will allow the construction of optimal response surfaces, ensuring a more accurate prediction compared to a single ANN with three outputs. The outcome of this step of the algorithm will be the predicted values of the three interdependent attributes.
- ***Step 2.*** Three new datasets are formed by replacing the initial independent attributes of the problem with new ones, i.e., the three predicted attributes obtained from the first step of the algorithmic implementation of the developed cascade model. Each of the three datasets will differ only in the output signal or dependent attribute. Hence, each of the three datasets will contain its own output - one of the three output attributes to be predicted and which, for the training sample, are specified from the outset.
- ***Step 3.*** The prediction process, that includes the selection of optimal parameters using the method of differential evolution, is performed based on three new GRNNs using the corresponding new datasets from the previous step. This approach, once again, ensures the construction of an optimal response surface for each of the three sought output signals, whereas the new set of inputs for each dataset guarantees consideration of the interdependencies between the predicted output signals. The obtained values will be the sought solutions to the posed problem.

## Modelling and results

This section describes the modelling of the designed GRNN-based cascade ensemble model. Performance evaluation metrics of the developed model are provided, optimal parameters of its operation are determined, and the obtained results are summarised for the three different zones of the investigated bridge (see Fig. 3).

### Modelling

To implement the proposed modelling procedure, a custom software product using the Python programming language was developed. All numerical experiments were conducted on a computer with the following specifications: MacBook Air, Apple M2, 16 GB RAM. The collected representative training dataset did not contain any missing values, anomalies, or outliers. Therefore, no data-cleaning procedures were implemented. However, the datasets were normalised using the MinMaxScaler [30]. Application of the developed model was conducted using a training dataset that was developed using numerical analyses on several variations, i.e. different tendon loss scenarios, of a finite element model of an actual prestressed concrete balanced cantilever bridge. The training dataset contained 81 vectors. The test dataset on real bridge state identification has 5 vectors.

Input parameters were:

1. Vertical Drift Ratio of Zone 1
2. Vertical Drift Ratio of Zone 2
3. Vertical Drift Ratio of Zone 3
4. Concrete Young’s Modulus

Outputs:

1. Tendon Losses in Zone 1
2. Tendon Losses in Zone 2
3. Tendon Losses in Zone 3

### Performance indicators

For evaluating the effectiveness of the proposed GRNN-based cascade ensemble model a series of metrics were utilised, including [31, 32]:

- Coefficient of determination (R^2^):
  4 $${R}^{2}=1-\frac{\sum_{i=1}^{N}{\left({y}_{i}^{true}-{y}_{i}^{pred}\right)}^{2}}{\sum_{i=1}^{N}{\left({y}_{i}^{true}-\overline{{y }_{i}}\right)}^{2}},\text{ where }{y}_{i}= \frac{1}{N}\sum_{i=1}^{N}{y}_{i}^{true}$$
- Root mean square error (RMSE):
  5 $$RMSE=\sqrt{\frac{\sum_{i=1}^{N}{\left({y}_{i}^{true}-{y}_{i}^{pred}\right)}^{2}}{N}}$$
- Maximum residual error (MaxE):
  6 $$ME=max\left(\left|{y}_{i}^{true}-{y}_{i}^{pred}\right|\right)$$
- Median absolute error (MedAE):
  7 $$MedAE=median\left(\left|{y}_{i}^{true}-{y}_{i}^{pred}\right|, \dots ,\left|{y}_{N}^{true}-{y}_{N}^{pred}\right|\right)$$
- Mean absolute error (MAE):
  8 $$MAE=\frac{1}{N}\sum_{i=1}^{N}\left|{y}_{i}^{true}-{y}_{i}^{pred}\right|$$
- Mean square error (MSE):
  9 $$MSE=\frac{1}{N}\sum_{i=1}^{N}{\left({y}_{i}^{true}-{y}_{i}^{pred}\right)}^{2}$$
- Mean absolute percentage error (MAPE):
  10 $$MAPE=\frac{1}{N}\sum_{i=1}^{N}\frac{\left|{y}_{i}^{true}-{y}_{i}^{pred}\right|}{{y}_{i}^{true}}$$
  where ${y}_{i}^{true}$ is the true value for *i*th observation, and ${y}_{i}^{pred}$ is the obtained (predicted) value for *ith* observation, for $i=1,N$ where $N$ is the total number of dataset observations.

The analysis of the results obtained, based on the combined utilisation of the metrics outlined in Eqs. (4)–(10), will provide a comprehensive assessment of the effectiveness of the proposed method. This comprehensive evaluation approach allows for a thorough understanding of the model's performance across various aspects, encompassing accuracy, robustness, and generalisation capabilities. By integrating multiple metrics, insights into how well the model performs under different conditions, providing a more holistic perspective on its overall performance [31] could be gained. Furthermore, the adopted evaluation approach enables informed decisions to be made regarding the suitability and reliability of the proposed GRNN-based cascade ensemble model for the given task of non-destructive bridge damage state identification.

### Results

As mentioned in the previous section, the effectiveness of the GRNN, that forms the basis of the designed GRNN-based cascade ensemble model for non-destructive bridge damage state identification, largely depends on the value of a single parameter, that is the spread of the Gaussian activation function (σ) [33]. To determine this parameter, the differential evolution method was utilised. Table 3 in Appendix provides the optimal values of σ for solving the addressed task.

It is essential to emphasise the critical role of parameter tuning, particularly with regard to the σ, in achieving an optimal performance of the model. The use of differential evolution methods allows for the efficient exploration of the parameter space, ensuring that the model is appropriately configured for the task at hand. Furthermore, by examining the results presented in Table 2, one may assess the performance of the model across various evaluation metrics, providing a comprehensive understanding of its effectiveness in identifying non-destructive bridge damage states. These results serve as a valuable guidance for further refinement and optimisation of the model, ultimately contributing to its applicability and reliability in real-world scenarios.

**Table 2:** Results of the designed GRNN-based cascade ensemble model

Performance indicators	Values of the performance indicators		
	*Zone 1*	*Zone 2*	*Zone 3*
R^2^	0..87	0.99	0.85
RMSE	3.612	1.003	2.698
MaxE	6.335	1.902	4.326
MedAE	2.602	0.299	0.52
MAE	3.042	0.683	1.82
MSE	13.043	1.007	7.278
MAPE	0.07	0.025	0.328
Application time	0.075	0.06	0.188

Table 2 presents the results of the designed GRNN-based cascade ensemble model considering the performance indicators provided in Eqs. (4)–(10).

As evident by inspecting the results summarised in Table 2, the developed method demonstrates high accuracy metrics for solving the task of non-destructive bridge damage state identification. Specifically, the coefficient of determination of the developed model stands at 87%, 97%, and 93% respectively for Zone 1, Zone 2, and Zone 3 of the investigated bridge.

These high R^2^ and low error metrics underscore the efficacy of the proposed GRNN-based cascade ensemble model in accurately identifying the damage state (i.e., tendon losses) in each zone of the bridge deck. Such accuracy is crucial for determining the reliability and safety of bridge infrastructure based on monitoring data, since it allows early detection and assessment of potential structural issues and timely implementation of the required mitigation and strengthening measures.

## Comparison and discussion

To evaluate the effectiveness of the designed GRNN-based cascade ensemble model for non-destructive bridge damage state identification, a comparison of its performance with a number of existing methods was also conducted. Among them, comparisons with the following methods worth to be highlighted:

- Classical GRNN from [18]
- Extended-inputs GRNN from [33]
- Input-doubling method based on GRNN from [27]
- *KNN*-based method [34] and its application for solving the stated task by Kazantzi et al. [35] (the 3-kNN weighted method)

It should be noted that the Classical GRNN [18], the Extended-inputs GRNN [33], and the Input-doubling method based on GRNN [27] also utilise GRNN. All of them demonstrate high efficiency in solving various regression tasks, especially when analyzing a small dataset. However, considering the specific task, and in particular the need to predict three interdependent attributes of a given dataset, all of them can only be used as the initial step of the designed method (as weak regressors). A comparison of the proposed method with the application of *KNN*-based method [35] will be most informative since this method takes into account problem #3.

The modelling of the operation of all existing methods was conducted with the selection and utilisation of the optimal parameters. For the methods based on the use of GRNN [18, 27, 33], the search for the optimal value of the smooth factor was conducted using the method of differential evolution. The optimal operation parameters of the KNN-based method [34] were taken from the paper [35]. The values of the optimal parameters for all the investigated methods are summarised in Table 3 in Appendix.

**Fig. 6:**
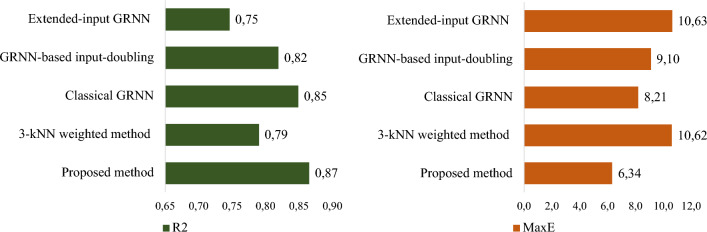
A comparative analysis of the efficacy of the proposed method against established approaches, utilizing R^2^ and MaxE for Zone 1

For enabling a detailed assessment of the operational accuracy of all the investigated methods, a comparison was conducted for the predictions related to the tendon losses in each zone separately. In particular, the prediction accuracy of the considered methods for the damage observed in Zone 1 based on the two most significant metrics, i.e., the R^2^ and the MaxE, are presented in Fig. 6.

By inspecting Fig. 6, it is evident that the least accurate results were obtained using the methods Extended-inputs GRNN [33] and the KNN-based [35] methods. For the Extended-inputs GRNN he aforementioned observation could be explained as follows: despite the high accuracy of this method in solving other tasks, for the case at hand, it makes predictions using a set of independent attributes without considering the existence of interdependencies between the three output parameters. Such an approach reduces the amount of necessary information for each of the mentioned methods, that consequently reduces the accuracy of the predictions. Moreover, this method significantly expands the space of input data of the problem, which reduces its generalisation properties. Therefore, the accuracy in this case is the lowest. The 3-kNN weighted method [35] demonstrates higher predicted effectiveness for the desired attribute in Zone 1 based on (4), since it takes into account the interdependencies between the three desired attributes. However, it shows significantly worse results compared to the Classical GRNN [18], and the Input-doubling method based on GRNN [27] methods. This can be explained by the fact that the method from KNN-based method [35] is a significantly simpler version compared to the methods [27, 33].

Classical GRNN [18], and input-doubling method [27] demonstrate significantly better results than the previous two methods. However, they, like the Extended-inputs GRNN [33], do not take into account the specifics of the considered task, namely the interdependence of the three sought-after attributes.

The highest prediction accuracy for the desired attribute in Zone 1 was achieved using the designed GRNN-based cascade ensemble model. Specifically, an 8% higher accuracy was achieved based on R^2^ (4) compared to the 3-kNN weighted method [35] for solving the given task.

**Fig. 7:**
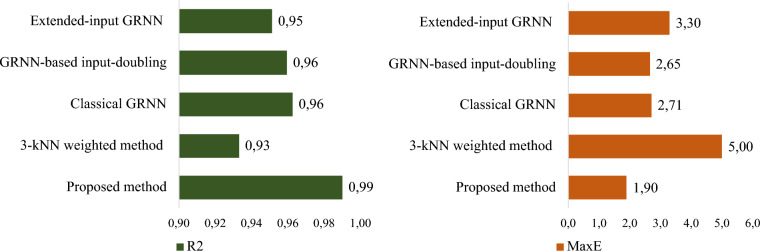
A comparative analysis of the efficacy of the proposed method against established approaches, utilizing R^2^ and MaxE for Zone 2

The prediction accuracy of the investigated methods for the desired value in Zone 2 based on (4) and (6) is illustrated in Fig. 7.

As seen from Fig. 7, the accuracy of the classical GRNN [18], an extended-inputs GRNN [33], and input-doubling method [27], according to the coefficient of determination is quite high, reaching almost 96%. However, the high values of the maximum residual error (MaxE) indicate that the predictions are not successful for all elements of the test sample. This reduces the reliability of applying such methods to solve the given task.

Similarly to the previous case, the 3-kNN weighted method [35] demonstrates worse effectiveness in the prediction of the desired attribute for Zone 2 based on (6). In particular, the maximum error value has been increased by almost 2 times compared to all other investigated methods. In addition, the accuracy of this method based on (4) is significantly lower than all other methods. This is again explained by the simplicity of the method [35] compared to classical GRNN [18], and extended-inputs GRNN [33].

The highest prediction accuracy based on all investigated methods for Zone 2 was achieved using the proposed GRNN-based cascade ensemble model. Specifically, a 38% higher accuracy was achieved based on (4) compared to the 3-kNN weighted method [35] for solving the given task.

**Fig. 8:**
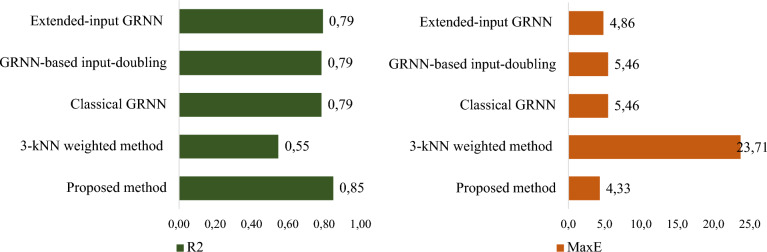
A comparative analysis of the efficacy of the proposed method against established approaches, utilizing R^2^ and MaxE for Zone 3

Finally, the results of comparing the forecast accuracy of the investigated methods for Zone 3 (Fig. 8) are presented.

As seen in Fig. 8, the 3-kNN weighted method [35] showed significantly unsatisfactory accuracy based on (4) and (6). Moreover, the existing methods, i.e., classical GRNN [18], an extended-inputs GRNN [33], and input-doubling method [27], demonstrate almost identical, quite low prediction accuracy for the desired attribute. This is confirmed by all performance indicators (4)–(10).

The highest prediction accuracy for the desired attribute for Zone 3 was achieved using the designed GRNN-based cascade ensemble model. Specifically, the coefficient of determination shows that a 30% higher accuracy was achieved compared to the method from the 3-kNN weighted method and a 6% higher accuracy compared to other methods.

In summary, it is important to note the following:

- The task addressed in this study, namely the prediction of three interdependent output attributes, is rather unconventional in the field of ML, and therefore there are very few methods available in the literature for its solution.
- The task becomes significantly more challenging when analysing a small dataset, as the application of existing ML methods is accompanied by many significant problems [27].
- To comprehensively evaluate the results of the developed model, including accuracy, reliability, and robustness, various performance indicators should be used collectively, as done in this study.
- Existing methods, i.e. classical GRNN [18], an extended-inputs GRNN [33], and input-doubling method [27], demonstrate relatively satisfactory prediction accuracy for all three desired attributes based on different performance indicators. However, existing methods do not consider the specifics of the task investigated, namely the interdependence of the three attributes to be predicted. This reduces the effectiveness of their application for solving the task at hand, essentially making them serve as weak predictors only in the first or last step of the designed GRNN-based cascade ensemble model.
- The 3-kNN weighted method [35], that considers the specifics of the task, shows a significant decrease in accuracy based on individual performance indicators compared to all other methods.
- Moreover, the 3-kNN weighted method [35] shows somewhat contradictory results when predicting the desired attribute for across the three different bridge zones. This is because it is a much simpler version of the classical GRNN method.
- Considering that the classical GRNN [18] demonstrates the highest prediction accuracy compared to extended-inputs GRNN [33], and input-doubling method [27], for all three zones, the authors made the right choice of this method as a weak predictor for constructing the new ensemble model.
- The GRNN-based cascade ensemble model proposed in this study is specifically designed to account for the interdependence between the three desired attributes and the need to analyse a small dataset.
- The designed GRNN-based cascade ensemble model demonstrates the highest prediction accuracy among all investigated methods, particularly based on the coefficient of determination. This allows for the practical application of this method.
- Additionally, the developed model shows the lowest values of maximum error among all the methods investigated. This is an indicator of its good prediction capabilities across the entire test sample and thereby increasing the reliability of using the proposed method for solving the problem of non-destructive bridge damage state identification.

Further research could be focused on achieving even higher accuracy of the designed GRNN-based cascade ensemble model. Pursuing this task can be done in the following main directions:

1. The use of other weak predictors as base models in the cascade ensemble is planned. Specifically, it is intended to improve the performance of the input-doubling method based on GRNN [27] by linearising the response surface, which should significantly increase its accuracy. Consequently, applying a more accurate version of this method at both levels of the developed cascade will enhance the prediction accuracy of the entire model.
2. The second direction of further research will involve the procedure of the formation of a new data sample after completing the first level of the cascade. In particular, expanding the input data space (in our case, the current sample contains only three independent attributes) according to Cover's theorem will increase the prediction accuracy at the second level of the cascade. This can be implemented by adding initial attributes of the new dataset or performing nonlinear input expansion using the Wiener polynomial or RBF functions [36, 37].
3. The third direction may involve an iterative procedure for refining the prediction at the third step of the method. Specifically, the three predicted values of the desired attributes after completing the third step of the cascade model can replace the data sample in the second step of the method. This may provide the opportunity to obtain more accurate predictions using a certain number of iterations.
4. The last direction for potential improvement in the accuracy of the designed GRNN-based cascade ensemble model is the combination of all three approaches described above.

## Conclusions

This paper aims to facilitate the rapid decision-making for ageing bridges impacted by climate change which accelerates their deterioration, leading to deflections and damage. Despite the availability of methods for rapid bridge assessment, very few of these methods leverage Machine Learning (ML) algorithms to predict displacements and link them to damage. Even fewer develop new ML algorithms to improve the prediction accuracy. The reason is that analyses are expensive to run and as a result only small datasets can be developed to train ML algorithms—bound to the time and resource limitations at the assessment stage. To address this challenge, this paper proposes the utilisation of a novel ML algorithm to enable rapid and accurate damage identification and characterisation.

First, a comprehensive tabular dataset was complied by simulating various plausible bridge damage scenarios using state-of-the-art numerical modelling. Next, the dataset was utilised as the training set for enabling non-destructive bridge damage state identification. This step also involved the development of a fit-for-purpose ML algorithm to improve the prediction accuracy. Specifically, a novel, two-level GRNN-based cascade ensemble model was introduced to predict three important interdependent output attributes (i.e., the tendon losses in the three bridge zones), addressing the challenge posed by the availability of a limited dataset. The study revealed that the new ML algorithm successfully tackles the unconventional task of predicting the three interdependent output attributes by utilising input data that can be relatively easily obtained by field measurements that require limited interaction with the asset. These developments present a groundbreaking solution in the fields of machine learning and bridge damage state identification. The optimisation of the proposed cascade model was accomplished through the utilisation of the differential evolution method, ensuring enhanced performance.

The effectiveness of the cascade model developed was validated by a real case-study involving a bridge, demonstrating superior performance in that it accurately identifies bridge damage states compared to existing methods. The GRNN-based cascade ensemble model exhibits high prediction accuracy, particularly for the available small datasets, thus showcasing its practicality in non-destructive bridge damage assessment. Furthermore, the model developed demonstrates minimal maximum error values across the entire test sample, thereby increasing the reliability of non-destructive bridge damage state identification.

Future research will focus on improving the developed cascade ensemble model through various means, including the exploration of options for expanding inputs, the development of an iterative procedure for specifying regression model parameters, and the enhancement of input-doubling methods based on GRNN as weak predictors. These efforts aim to increase the accuracy of non-destructive bridge damage state identification by machine learning methods, especially in view of small datasets. With regard to training data, these could be provided by monitoring sources, to enable more precise damage identification, as latent damage is not always identifiable during routine inspections.

## Data Availability

No datasets were generated or analysed during the current study.

## Appendix

See Table 3.

**Table 3:** Optimal parameters for all investigated methods

Method	Optimal parameters		
	*Zone 1*	*Zone 2*	*Zone 3*
Proposed method	σ1 = 0.09000888	σ1 = 0.088416428	σ1 = 0.037662066
	σ2 = 0.150033535	σ2 = 0.092188517	σ2 = 0.124612538
*KNN*-based method [35]	3 clusters	3 clusters	3 clusters
Classical GRNN [18]	σ = 0.037700764	σ = 0.088416428	σ = 0.037662522
Input-doubling method based on GRNN [27]	σ = 0.105310021	σ = 0.079040101	σ = 0.03766001
Extended-inputs GRNN [33]	σ = 0.097772416	σ = 0.130925064	σ = 0.078732229
